# Draft Genome Sequences of 64 Type Strains of 50 Species and 25 Subspecies of the Genus *Staphylococcus* Rosenbach 1884

**DOI:** 10.1128/MRA.00062-19

**Published:** 2019-04-25

**Authors:** Kevin Cole, Dona Foster, Julie E. Russell, Tanya Golubchik, Martin Llewelyn, Daniel J. Wilson, Derrick Crook, John Paul

**Affiliations:** a National Infection Service, Public Health England, London, United Kingdom; b Brighton and Sussex University Hospitals NHS Trust, Brighton, United Kingdom; c Division of Medicine, Brighton and Sussex Medical School, Falmer, United Kingdom; d Nuffield Department of Medicine, University of Oxford, Oxford, United Kingdom; e Department of Statistics, University of Oxford, Oxford, United Kingdom; f National Collection of Type Cultures, Public Health England, London, United Kingdom; g Wellcome Trust Centre for Human Genetics, University of Oxford, Oxford, United Kingdom; University of California, Riverside

## Abstract

Members of the genus *Staphylococcus* have been isolated from humans, animals, and the environment. Accurate identification with whole-genome sequencing requires access to data derived from type strains. We provide sequence data for type strains of 64 taxa in the genus that at the time of this writing have standing in the nomenclature.

## Abstract (precis)

Members of the genus *Staphylococcus* have been isolated from humans, animals, and the environment. Accurate identification with whole-genome sequencing requires access to data derived from type strains.

## ANNOUNCEMENT

The term “*Staphylococcus*” was introduced by Sir Alexander Ogston in reference to the bunch-of-grapes-like appearance that distinguishes these organisms from streptococci (1). Friedrich Rosenbach subsequently described “Staphylococcus pyogenes *aureus”* and “Staphylococcus pyogenes *albus”* (2). At the time of this writing, the genus includes 51 species plus additional subspecies. They are nonmotile, facultatively or obligatory anaerobic, Gram-positive cocci (3–5). Most are catalase positive (4–6). Members of the genus include human and animal pathogens and commensals, and they have been isolated from foodstuffs and environmental sources (7, 8). S. aureus is a well-known human pathogen, and the genome sequence of the type strain was published by Kim et al. in 2014 (9) and Shiroma et al. in 2015 (10). Other staphylococci are increasingly becoming recognized as clinically important (7, 11) and are being investigated accordingly (12, 13).

Here we give information for genome sequences of 64 type strains (Table 1) representing 50 species in the genus *Staphylococcus* that have standing in the nomenclature at the time of writing plus a number of subspecies. This catalogue of sequences can be employed as a resource for taxonomic study and to identify test isolates.

**TABLE 1:** Accessions and genomic characteristics of 64 type strains of the genus *Staphylococcus* Rosenbach 1884

Species	Strain	No. of total reads	No. of contigs	Contig *N*_50_ value (bp)	G+C content (%)	Depth (×)	Length (bp)	No. of genes
Staphylococcus agnetis Taponen et al. 2012	DSM 23656	1,978,702	164	38,640	34.6	66.4	2,491,359	2,514
Staphylococcus argensis Heß and Gallert 2015	DSM 29875	1,430,626	19	98,914	40.8	24.75	2,452,468	2,350
Staphylococcus argenteus Tong et al. 2015	DSM 28299	1,686,623	48	96,207	35.1	31.51	2,759,629	2,741
Staphylococcus arlettae Schleifer et al. 1985	NCTC 12413	1,992,335	33	66,907	36.2	26.57	2,665,344	2,600
Staphylococcus aureus subsp. *anaerobius* De La Fuente et al. 1985	DSM 20714	1,949,112	400	14,564	33.2	56.12	2,575,746	2,738
Staphylococcus aureus subsp. *aureus* De La Fuente et al. 1985	NCTC 08532	2,207,024	41	66,458	34.9	32.75	2,749,019	2,887
Staphylococcus auricularis Kloos and Schleifer 1983	NCTC 12101	1,517,960	103	39,462	37.6	38.94	2,201,622	2,416
Staphylococcus capitis subsp. *capitis* Bannerman and Kloos 1991	NCTC 11045	2,331,749	51	101,914	36	38.76	2,434,909	2,419
Staphylococcus capitis subsp. *urealyticus* Bannerman and Kloos 1991	DSM 6717	1,467,277	184	42,481	35.7	35.79	2,468,245	2,500
Staphylococcus caprae Devriese et al. 1983	NCTC 12196	2,647,473	101	62,212	33.6	62.25	2,606,761	2,551
Staphylococcus carnosus subsp. *carnosus* Probst et al. 1998	DSM 20501	1,442,326	70	247,399	37.7	90.42	2,434,039	2,447
Staphylococcus carnosus subsp. *utilis* Probst et al. 1998	DSM 11676	1,834,824	444	11,755	33.4	36.49	2,622,255	2,702
Macrococcus caseolyticus Schleifer et al. 1982	DSM 20597	1,614,157	122	46,917	35.5	70.8	2,171,833	2,341
Staphylococcus chromogenes Hájek et al. 1987	NCTC 10530	1,169,641	117	55,359	38.5	35.94	2,276,768	2,265
Staphylococcus cohnii subsp. *cohnii* Kloos and Wolfshohl 1991	NCTC 11041	1,727,741	69	107,943	34.9	26.83	2,637,875	2,588
Staphylococcus cohnii subsp. *urealyticus* Kloos and Wolfshohl 1991	DSM 6718	1,780,154	189	26,500	33.8	49.2	2,670,427	2,647
Staphylococcus condimenti Probst et al. 1998	DSM 11674	1,493,311	211	32,221	35.8	23.6	2,615,688	2,604
Staphylococcus delphini Varaldo et al. 1988	NCTC 12225	1,499,070	227	42,122	37.1	52.47	2,751,077	2,716
Staphylococcus devriesei Supré et al. 2010	CCUG 58238	2,004,126	129	49,574	35.6	44.18	2,379,883	2,392
Staphylococcus epidermidis Kloos and Schleifer 1975	NCTC 11047	1,852,043	62	55,347	35.8	38.72	2,442,385	2,438
Staphylococcus equorum subsp. *equorum* Place et al. 2003	NCTC 12414	1,523,028	93	44,027	33.2	87.8	2,350,071	2,590
Staphylococcus equorum subsp. *linens* Place et al. 2003	DSM 15097	1,911,830	101	55,558	34	45.91	2,768,268	2,739
Staphylococcus felis Igimi et al. 1989	DSM 7377	1,572,866	244	24,395	36.1	45	2,409,047	2,389
Staphylococcus fleurettii Vernozy-Rozand et al. 2000	DSM 13212	1,811,506	156	38,940	31.7	90.36	2,473,007	2,489
Staphylococcus haemolyticus Kloos and Schleifer 1975	NCTC 11042	1,975,151	92	55,720	36	35.24	2,472,399	2,466
Staphylococcus hominis subsp. *hominis* Kloos et al. 1998	NCTC 11320	2,793,372	30	144,174	39.1	47.59	2,204,528	2,214
Staphylococcus hominis subsp. *novobiosepticus* Kloos et al. 1998	CCUG 42399	2,349,019	166	35,627	33.4	54.32	2,422,390	2,498
Staphylococcus hyicus Hájek et al. 1987	CCUG 6509	2,111,770	134	37,537	37.7	91.88	2,633,558	2,222
Staphylococcus intermedius Hájek 1976	NCTC 11048	2,212,778	176	51,566	37.8	41.45	2,801,199	2,797
Staphylococcus kloosii Schleifer et al. 1985	NCTC 12415	2,000,256	134	36,209	33	74.73	2,607,914	2,642
Staphylococcus lentus Schleifer et al. 1983	NCTC 12102	3,158,427	79	86,117	32.7	93.32	2,546,437	2,579
Staphylococcus lugdunensis Freney et al. 1988	NCTC 12217	2,272,326	20	94,638	39.8	35.15	2,519,514	2,409
Staphylococcus lutrae Foster et al. 1997	DSM 10244	1,461,603	200	41,148	37.8	42.6	2,429,515	2,354
Staphylococcus massiliensis Al Masalma et al. 2010	CCUG 55927	2,070,218	212	32,379	35	41.69	2,348,540	2,305
Staphylococcus microti Nováková et al. 2010	DSM 22147	1,582,916	191	38,659	39.1	53.13	2,409,945	2,429
Staphylococcus muscae Hájek et al. 1992	DSM 7068	1,986,642	183	32,200	37.4	63.32	2,049,263	2,075
Staphylococcus nepalensis Spergser et al. 2003	DSM 15150	2,186,623	412	13,339	32.9	50.09	2,860,226	2,790
Staphylococcus pasteuri Chesneau et al. 1993	DSM 10656	1,952,357	233	25,197	31.4	89.61	2,605,275	2,277
Staphylococcus petrasii subsp. *croceilyticus* Pantůček et al. 2013	CCUG 62728	1,665,214	147	36,044	34.6	55.76	2,380,571	2,346
Staphylococcus petrasii subsp. *jettensis* De Bel et al. 2014	CCUG 62657	1,923,933	266	23,568	34.7	45.4	2,709,801	2,730
Staphylococcus petrasii subsp. *petrasii* Pantůček et al. 2013	CCUG 62727	2,674,384	139	46,072	34.1	58.57	2,486,611	2,508
Staphylococcus petrasii subsp. *pragensis* Švec et al. 2015	DSM 102853	2,071,432	493	11,863	33	87.81	3,094,570	3,040
Staphylococcus pettenkoferi Trülzsch et al. 2007	CCUG 51270	1,933,064	122	62,580	37.2	46.82	2,455,272	2,399
Staphylococcus piscifermentans Tanasupawat et al. 1992	DSM 7373	1,828,752	147	37,718	37	91.44	2,711,458	2,470
Staphylococcus pseudintermedius Devriese et al. 2005	CCUG 49543	1,307,526	245	32,222	37.3	33.7	2,507,403	2,482
Staphylococcus pulvereri Zakrzewska-Czerwińska et al. 1995	DSM 9930	1,085,309	252	21,767	32.8	91.9	2,507,841	2,321
Staphylococcus rostri Riesen and Perreten 2010	DSM 21968	1,857,275	167	39,749	37.6	72.78	2,341,022	2,342
Staphylococcus saccharolyticus Kilpper-Bälz and Schleifer 1984	NCTC 11807	1,735,439	93	46,092	34.5	91.58	2,462,952	2,724
Staphylococcus saprophyticus subsp. *bovis* Hájek et al. 1996	CCUG 38042	1,774,718	289	16,602	32.9	48.26	2,709,959	2,757
Staphylococcus saprophyticus subsp. *saprophyticus* Hájek et al. 1996	NCTC 7292	2,626,126	123	46,677	33.3	90.52	2,589,171	2,567
Staphylococcus schleiferi subsp. *coagulans* Igimi et al. 1990	DSM 6628	1,893,687	155	54,157	36.2	41.27	2,443,567	2,378
Staphylococcus schleiferi subsp. *schleiferi* Igimi et al. 1990	NCTC 12218	3,174,090	98	67,466	37.6	90.54	2,896,454	2,368
Staphylococcus schweitzeri Tong et al. 2015	DSM 28300	1,956,713	52	65,148	35.5	37.08	2,743,713	2,725
Staphylococcus sciuri subsp. *carnaticus* Kloos et al. 1997	CCUG 39509	1,454,961	182	40,393	33.6	51.84	2,877,673	2,983
Staphylococcus sciuri subsp. *rodentium* Kloos et al. 1997	CCUG 37923	925,469	82	62,754	36.6	91.96	2,449,200	2,919
Staphylococcus sciuri subsp. *sciuri* Kloos et al. 1997	NCTC 12103	2,205,792	37	123,278	38.7	32.97	2,768,322	2,760
Staphylococcus simiae Pantucek et al. 2005	CCUG 51256	2,569,797	159	45,657	34.3	77.79	2,598,081	2,523
Staphylococcus simulans Kloos and Schleifer 1975	NCTC 11046	1,359,349	99	84,647	37.2	48.33	2,735,408	2,699
Staphylococcus stepanovicii Hauschild et al. 2012	DSM 26319	1,676,662	140	42,374	35.1	36.62	2,406,018	2,468
Staphylococcus succinus subsp. *casei* Place et al. 2003	DSM 15096	1,816,917	169	29,746	34.8	56.18	2,871,374	2,802
Staphylococcus succinus subsp. *succinus* Place et al. 2003	DSM 14617	1,805,866	339	18,694	32.2	29.39	2,786,115	2,764
Staphylococcus vitulinus Webster et al. 1994	DSM 15615	1,414,448	259	23,873	32.5	57.23	2,595,808	2,674
Staphylococcus warneri Kloos and Schleifer 1975	NCTC 11044	1,997,389	31	115,611	38	35.2	2,401,190	2,353
Staphylococcus xylosus Schleifer and Kloos 1975	NCTC 11043	1,802,579	226	25,849	34.4	51.79	2,725,582	2,615

Organisms were obtained from the National Collection of Type Cultures, United Kingdom (NCTC), Deutsche Sammlung von Mikroorganismen und Zellkulturen, Germany (DSMZ), and Culture Collection, University of Göteborg, Sweden (CCUG).

Isolates were cultured on a Columbia agar and horse blood (CBA) plate (Oxoid Ltd., Basingstoke, UK) and incubated aerobically or anaerobically at 35°C overnight. Bacterial biomass was scraped from cultured plates for DNA extraction. DNA was extracted and purified with a QuickGene DNA tissue kit (AutoGen, Holliston, MA, USA). Extracted DNA was prepared for sequencing following NexteraXT (Illumina, San Diego, CA, USA) protocols and sequenced as 150-bp paired-end reads on the Illumina MiSeq platform. Reads were stripped of adaptors with BBDuk 34.38 (14). Illumina reads were quality controlled by calling bases only when 5 or more were present at a position and supported by at least 1 high-quality read in forward and reverse directions. Reads were assembled using Velvet 1.2.10 (15) with kmer size and coverage estimated with VelvetOptimiser 2.1.7 (15). To ensure assembly quality, at least 97% of the total assembly was required to be in contigs larger than 1 kb. Draft genome sequences were annotated with Prokka 1.12 (16).

Genome sizes varied between 2.1 million and 3.1 million base pairs across the genus, with G+C contents ranging from 31.4% to 40.8%. The genome sequences contained a range of 2,075 to 3,040 annotated genes.

### Data availability.

All sequences discussed here have been deposited in GenBank as BioProject number PRJNA339206. Table 1 lists individual accession numbers by taxon. These Sequence Read Archive deposits can be found under the study number SRP093495.
